# Colorimetric and Label-Free Optical Detection of Pb^2+^ Ions via Colloidal Gold Nanoparticles

**DOI:** 10.3390/bios13080819

**Published:** 2023-08-15

**Authors:** Jasmin A. Flowers, Monique J. Farrell, Gugu Rutherford, Aswini K. Pradhan

**Affiliations:** 1 Boston Scientific, Maple Grove, MN 55311, USA; 2 FTL at Northrop Grumman, Washington, DC 20088, USA; 3 NASA Langley Research Center, Langley Boulevard, Hampton, VA 23681, USA; 4 Department of Physics, Hampton University, Hampton, VA 23668, USA

**Keywords:** gold nanoparticles, surface plasmons, calorimetric detection of metal ions

## Abstract

The detection of the lead heavy metal (Pb) in water is crucial in many chemical processes, as it is associated with serious health hazards. Here, we report the selective and precise colorimetric detection of Pb^2+^ ions in water, exploiting the aggregation and self-assembly mechanisms of glutathione (GSH)-functionalized gold nanoparticles (GNPs). The carboxyl functional groups are able to create coordination complexes with Pb^2+^, inducing aggregation amongst the GSH-GNPs in the presence of Pb^2+^ due to the chelation of the GSH ligands. The resulting aggregation amongst the GSH-GNPs in the presence of Pb^2+^ increases the aggregate size depending on the available Pb^2+^ ions, affecting the plasmonic coupling. This causes a substantial shift in the plasmon wavelength to a longer wavelength side with increasing Pb^2+^ concentration, resulting in a red-to-blue colorimetric or visual change, enabling the instant determination of lead content in water.

## 1. Introduction

Among other heavy metals, lead has caused extensive contamination and health problems in many parts of the world as it is extremely harmful to the environment and severely detrimental to human health, even in low concentrations. It is a cumulative toxin that affects multiple body systems [1]. Specifically, due to bioaccumulation over time, even low concentrations of lead can have serious health effects, including death [1,2]. Generally, Pb disastrously contaminates city water due to hazardous waste disposal in local river water [3,4,5]. The contamination of drinking water with Pb caused by leaded water distribution systems and poisoning from degrading lead pipes and industrial waste disposal in rivers are pervasive problems and urgently need to be addressed [3]. The detection of Pb content in water remains a task of paramount importance. Atomic absorption spectrometry, inductively coupled plasma atomic emission spectrometry, and differential pulse anodic stripping voltammetry provide low limits of detection of around 1 ppb [5]. Unfortunately, these techniques, though highly sensitive, are also extremely sophisticated, costly, and, therefore, are not easily accessible. Another downside is that these sophisticated analytical techniques are not suitable for on-site applications. By developing a detection method that is quick, simple, cheap, and sufficiently sensitive, the great demand around the world would be met, and the method could also replace the current conventional analytical and expensive methods.

Here, we discuss the ability to detect lead by exploiting the mechanisms of the aggregation and self-assembly of GSH-GNPs. For the past decade, the field of environmental monitoring has seen nanoparticles, specifically gold, being utilized as functional probes for analyzing toxins, metal ions, and inorganic and organic pollutants [6,7]. Exploiting the properties of GNPs as functional probes provides the capability to use them both as colloidal and substrate-based sensors. Providing a quick and simplistic detection system could create the ability to test water worldwide, even in remote locations, to determine its viability. The method of using a GNP probe as a basis for the detection of Pb ions shows promise. We demonstrate a simple, quick, and selective colloidal colorimetric detection method for Pb^2+^ using highly stable, GSH-functionalized GNPs approximately as small as 15 nm in diameter.

## 2. Procedure and Methodology

The procedure for GNP synthesis in this work was based on a more recent method by Graber et al. [8,9], due to its simple, quick, and cheap synthesis. The materials used were gold (III) chloride trihydrate, barium chloride, copper (II) sulfate anhydrous, and cadmium acetate hydrate purchased from Sigma Aldrich. Sodium citrate, iron (II) chloride tetrahydrate, nickel (II) acetate hydrate, manganese (II) acetate anhydrous, zinc acetate anhydrous, calcium nitrate tetrahydrate, cobalt (II) acetate tetrahydrate, chromium (III) nitrate nonahydrate, and magnesium fluoride were purchased from Alfa Aesar. High-purity-grade L-glutathione was obtained from Amresco. All chemicals were used without further purification and were diluted using ultra-pure Millipore water (>18 MΩ) and deionized (D.I.) water. All glassware was cleaned vigorously with 3.5 M HCl, rinsed with copious amounts of milli-Q water, and allowed to dry before use.

The GNPs were synthesized via a quick and simple hot injection method. A solution of approximately 1 mM gold (III) chloride trihydrate was made by dissolving 40 mg in 100 mL of water. Note that the GNPs were synthesized with D.I. water and with milli-Q water to enable comparisons. The solution was allowed to heat to approximately 100 °C while being stirred, and this was the growth temperature used for GNPs here. Once it reached a low boil, a 39 mM sodium citrate (115 mg; 10 mL of water) solution was added. The solution was stirred for 10 min, resulting in a color change from pale yellow to a dark red wine color. After 10 min, the reaction was quenched by placing the beaker in an ice bath for 5 min while swirling the solution. To increase the working volume as well as aid in the cooling process, the solution was diluted 1:1, resulting in 200 mL of GNPs. To modify the surfaces of the GNPs, we chose glutathione (GSH) as our receptive ligand for Pb^2+^ detection. Using milli-Q water as the solvent, 19 mM GSH was made. To functionalize the GNPs, we briefly added 100 µL of 19 mM GSH to 800 µL of GNPs in a PMMA cuvette at room temperature. GSH is a non-protein tripeptide made up of cysteine, glycine, and glutamate amino acids. It is cheap, making it a very attractive candidate for surface and electrode modification and for the synthesis of monolayer-protected nanoparticles. The thiol group stemming from the cysteine amino acid has a high affinity towards metals [10,11], in particular gold, resulting in S-Au covalent bond formation [12]. After the addition of GSH to freshly prepared citrate-stabilized GNPs, their surfaces can be modified by GSH through the aforementioned S-Au covalent bond. GSH has two free –COOH groups and a –NH^2^ group to provide a hydrophilic interface and a handle for further reactivity with heavy metal ions [13]. At pH 8, the -NH^2^ group is protonated to -NH^3+^ and, as a result, -COO^-^ is the only binding site which is known to bind strongly to Pb^2+^ [14]. Metal ions like Fe^2+^, Cd^2+^, and Zn^2+^ are known to bind to the amino group in GSH, thus encouraging a pH of 8, where the amino group is made unavailable and the –COO^-^ is available for binding [14]. Incorporating the resulting zwitterionic form makes our system ideal for Pb^2+^ detection. To functionalize the GNPs, 100 µL of 19 mM GSH was added to 800 µL of GNPs at room temperature in the cuvette. After functionalization with GSH, the GNPs’ aggregation behavior was monitored in the presence of Pb^2+^ at room temperature.

The experimental trials were created by adding 900 µL of GSH-GNPs and 120 µL of 1 M NaCl to 120 µL of varying concentrations of Pb^2+^. After addition, the solution was sufficiently mixed by via pipetting it into a cuvette followed by centrifuging in order to ensure particle attestation with GSH. At high ionic strength, Pb^2+^ induced an immediate color change from red to blue which corresponded to changes in absorbance. This change in absorbance was measured using a LAMBDA 950 UV/Vis/NIR spectrometer from PerkinElmer. A time study was conducted from 0–60 min for each concentration of Pb^2+^ to monitor the progression of aggregation over time. The selectivity of the system for Pb over various other metal ions (Fe^2+^, Zn^2+^, Ba^2+^, Ni^2+^, Ca^2+^, Cr^3+^, Mg^2+^, Mn^2+^, Cu^2+^, and Cd^2+^) was investigated under the same conditions as previously stated. A concentration of 100 µM of each metal ion was tested and compared to 50 µM of Pb^2+^. SPR magnitude changes were investigated when initially mixed and after 10 min of incubation.

## 3. Results and Discussion

In the presence of Pb^2+^, GSH-GNPs undergo aggregation due to the formation of a chelating complex [15,16]. After GSH functionalization of the GNPs, the carboxyl functional groups are available to create coordination complexes with Pb^2+^. These coordination bonds are what induce aggregation of GSH-GNPs in the presence of Pb^2+^ due to the chelation of the GSH ligands [10,11]. Due to this mechanism of particle–particle coupling or plasmonic coupling, a substantial shift in the plasmon band energy to a longer wavelength occurs, resulting in a red-to-blue color change.

The shorter wavelengths, corresponding to a high energy, of light are only able to excite the localized SPR of smaller GNPs, and the longer wavelengths, corresponding to a low energy, of light are able to excite the localized SPR of larger GNPs. Therefore, small GNPs absorb shorter wavelengths, whereas large GNPs absorb longer wavelengths. This explains the shift in the SPR band to the right as the GNPs grow in size, resulting in the change of color from red to blue. When the GNPs aggregate, the distance between each particle decreases. This is where near-field coupling begins to dominate and produces a strong enhancement of the localized electric field within the inter-particle spacing. These enhanced electronic fields are confined within a very small space spanning the circumference of the GNPs and decay exponentially [17,18]. This mechanism proves highly beneficial because it exploits the local SPRs found on spherical nanoparticles to detect Pb^2+^ due to the change in energy of the GNPs.

**Figure 1:**
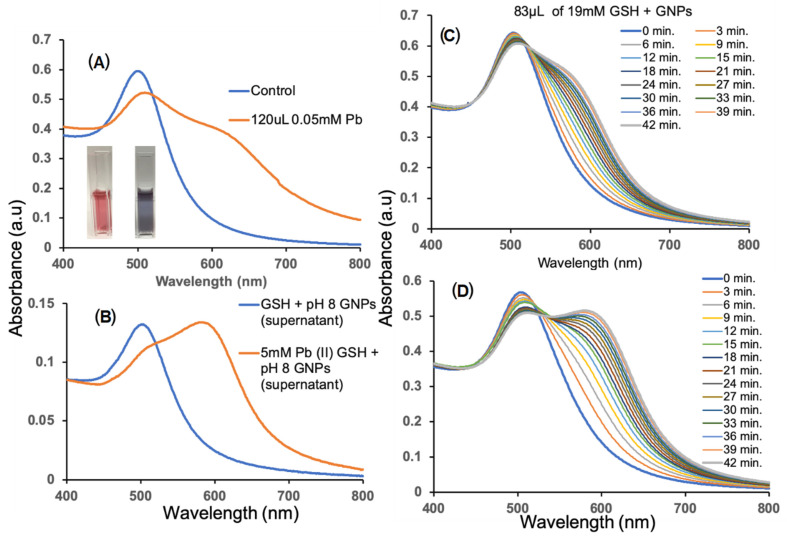
UV-Vis spectra and pictures of GSH-GNPs in the (**A**) absence and (**B**) presence of 0.05 mM Pb^2+^ of supernatant solutions at pH 8 and GSH-GNPs with and without addition of 5 mM Pb^2+^. The inset in (**A**) shows the colorimetric display with addition of 0.05 mM Pb^2+^ of supernatant solutions compared to the control. (**C**) UV-Vis spectra of 83 µL of 19 mM GSH added to GNPs to make up the control investigated over 42 min and (**D**) UV-Vis spectra of 0.05 mM Pb^2+^ added to GSH-GNPs over a 42 min timeframe.

A substantial shift in the plasmon band energy to a longer wavelength occurs, resulting in a red-to-blue color change as exhibited in Figure 1A,B in the presence of 0.05 mM Pb^2+^ of supernatant solutions of pH 8 and GSH-GNPs with and without addition of 5 mM Pb^2+^. Figure 1C,D shows the UV-Vis spectra of 83 µL of 19 mM GSH added to GNPs to make up the control, investigated over 42 min, and for 0.05 mM Pb^2+^ added to GSH-GNPs over a 42 min timeframe. Using the cleaned system, we employed a similar visual detection technique to determine the cleaned system’s capability to detect Pb^2+^. There have been reports [19] based upon the rate of aggregation in the presence of Pb^2+^ due to an unstable control. Though our control was aggregating at a similar rate when Pb^2+^ was added, we were unable to reach the same concentration sensitivity. Therefore, we explored ways to stabilize our control by altering our GSH solution to achieve a sufficient Pb^2+^ concentration sensitivity. First, we changed the amount of GSH used to functionalize the GNPs by reducing the volume of solution with a set concentration added to the 800 µL of GNPs. As expected, the lower the volume of GSH added, the more visually stable the control seemed. This is probably in part due to the pH of the GSH and the GNPs, which were 2.93 and 5.8, respectively. At an acidic pH, the functional groups of GSH are highly protonated, creating an overall positively charged molecule which is attracted to the negatively charged surface of the GNPs. The lower the amount of GSH molecules means the lower the amount of ligands to bind multiple GNPs together, ultimately meaning less aggregation or a slower rate of aggregation for the control. The volumes tested were 50, 60, 70, 80, 85, and 90 µL of 19 mM GSH. We noticed a slight color change with 85 µL and no color change with 80 µL immediately upon addition. With this in mind, 83 µL of 19 mM GSH was used to investigate the new control stability in a 42 min time study with measurements taken every 3 min, Figure 1C. This still did not provide the stable control we desired, as seen in the broadening and shifting of the SPR peaks in the absorbance spectra, indicative of aggregation and color change. Though the control was not ideal, we proceeded with the Pb^2+^ detection investigation to determine the control’s potential. A 42 min time study with 0.05 mM Pb^2+^ (Figure 1D) and a 20 h time drive absorbance study of the control and the lowest Pb^2+^ concentration (0.00001 mM Pb^2+^) were conducted. Similar to work by Zhong et al. [19], we investigated our control’s ability to detect Pb^2+^ based on the rate of aggregation. The control changed from red to purple within 10 min. When Pb^2+^ was added, a quicker rate of aggregation was noticed within the 10 min timeframe of the color change from red to blue. With this mechanism in mind, we sought to determine if we had achieved a similar aggregation rate, but within a longer timeframe.

**Figure 2:**
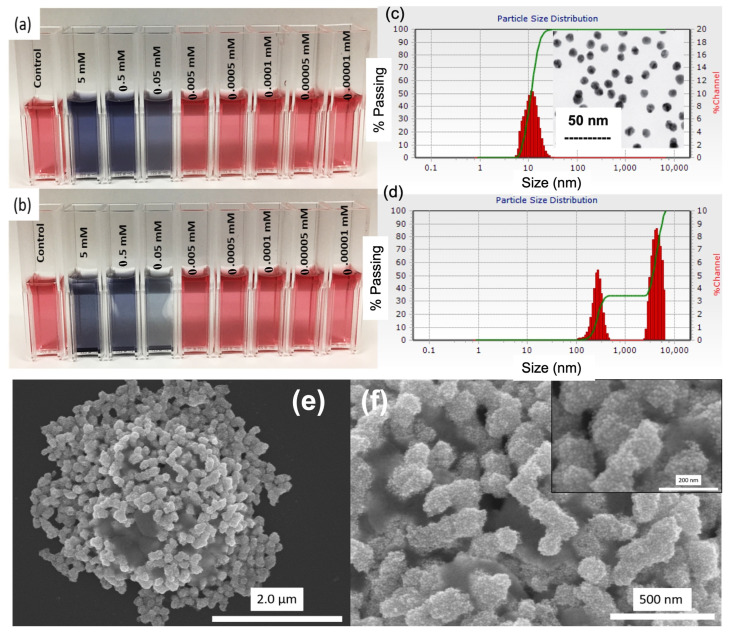
Images of colloidal solutions of GSH-GNPs at (**a**) 0 min and (**b**) 60 min of Pb^2+^ detection at various concentrations. Dynamic light scattering (DLS) data showing particle size distribution of control (Au NPs) (**c**) before and (**d**) after the addition of 120 µL of 0.05 mM Pb^2+^. (**e**) FESEM image of GSH-GNP aggregation induced by 50 µM Pb^2+^, and (**f**) enlarge scale of GSH-GNP aggregation induced by 50 µM Pb^2+^. Inset of (**c**) shows the FESEM image of Au NPs and inset of (**f**) shows the GSH-GNP aggregation induced by Pb^2+^ ions.

With a stable control and the successful colorimetric detection of Pb^2+^, we evaluated the detectable minimum concentration of Pb^2+^ in aqueous solution by color change. We added Pb^2+^ with concentrations of 5–0.00001 mM into the control. Figure 2a,b shows images of the decreasing concentrations of Pb^2+^ at 0 min and 60 min. The lower concentrations (0.0005 mM, 0.0001 mM, 0.00005 mM, and 0.00001 mM) do not follow the assumed trend. That is, as the concentration of Pb^2+^ decreases, we expect the peak width to decrease and revert back to the characteristic magnitude of the control. However, it does not, which indicates that the system is not able to properly discriminate between the low concentrations using UV-Vis as a characterization method. Limits of detection (LOD) are reported to be from 20 ppb [13] to 100 ppt [15]. However, they were not able to show visual detection at such low concentrations. Further optimization of this system with the goal of true visual detection of lower concentrations of Pb^2+^ would create the possibility of a competitive system that is quick and simple to make and detects trace levels of Pb^2+^ within 10 min.

A clear size distribution of the GSH-GNPs between 500 and 5000 nm is observed in the presence of Pb^2+^, as shown in Figure 2c–f, and this size distribution determines the plasmon frequency shift depending on the GNP aggregate size. A GNP-based simple colorimetric and ultrasensitive DLS assay was reported for the selective detection of Pb^2+^ [15]. A sensitivity down to 100 ppt was reported using DLS as a qualitative method of detection. Visually, however, they were only able to see a color change down to 1 ppm. Upon further optimization of our system, it is expected that we will see comparable results to these reported results. In addition to this, we provide a much better GNP synthesis method in terms of speed and stability of the product, as well as a faster detection timeframe.

It is clear that as the volume of Pb^2+^ increases, the SPR peak changes, attributed to the coupled plasmon absorbance of the GNPs in close contact. As the light in the UV-vis passes through the cuvette containing the solution, some is absorbed, and some is transmitted. The larger particle sizes within the solution correspond to longer wavelengths due to the changes triggered in the localized SPRs. This is why we observe broadened and shifted absorbance peaks. FESEM images help explain this visually. Figure 2e,f shows images of as-synthesized GNPs, GNPs functionalized with GSH, 1 M NaCl added to GSH-GNPs, and GNPs in the presence of Pb^2+^. The large aggregates cause a change in the localized SPRs of the individual GNPs due to coupling as well as the overall energy of the aggregate, which is reflected in the corresponding absorbance spectra. This large size correlates to the blue color we see in the system in the presence of Pb^2+^. Based on the obtained images from FESEM, the size of the as-synthesized D.I. GNPs averaged around 15 nm. This corresponds to the 500 nm absorbance wavelength characteristic in their SPR. As previously explained, the smaller size and consistency in shape of the GNPs correlate to lower wavelengths in the absorbance spectra. From this, we can confirm the synthesis of high-energy, uniformly spherical GNPs monodispersed in solution.

The size and shape of the as-synthesized GNPs create an ideal platform for colorimetric detection of Pb^2+^ due to their high surface area-to-volume ratio. The GNPs’ SPR frequency is highly sensitive to the refractive index/dielectric nature of their interface with the environment surrounding them [20]. Changing this environment will also shift the SPR frequency [21,22], thus enabling us to visually detect Pb^2+^ via colorimetric change. In addition to this, GNPs’ large surface areas enables them to be modified easily to become probes using thiolated or disulfide-modified ligands, electrostatic interactions, antibody–antigen associations, or streptavidin–biotin binding, as seen in many publications [13,23,24].

**Figure 3:**
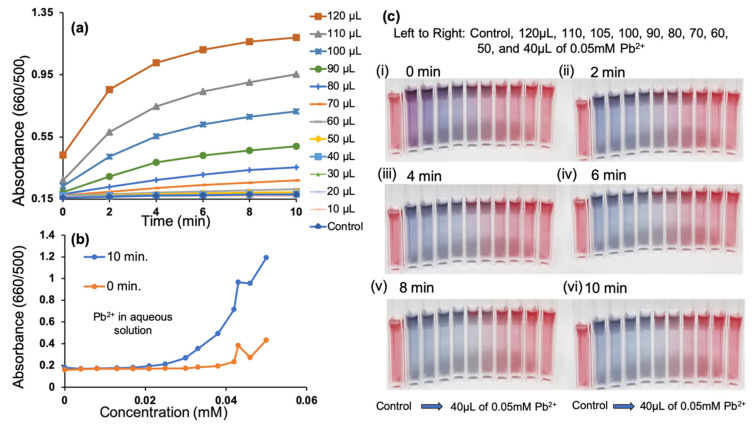
(**a**) Plots of the time-dependent absorption ratio (A_660/500_) over 10 min for various volumes of 0.05 mM Pb^2+^ added to the control. (**b**) A_660/500_ vs. various concentrations of Pb^2+^ in the range of 0.004–0.05 mM. (**c**) Images depicting the presence of decreasing volumes of 0.05 mM Pb^2+^ solution at (**i**) 0 min, (**ii**) 2 min, (**iii**) 4 min, (**iv**) 6 min, (**v**) 8 min, and (**vi**) 10 min. From left to right: control, 120 µL, 110 µL, 105 µL, 100 µL, 90 µL, 80 µL, 70 µL, 60 µL, 50 µL, and 40 µL of a 0.05 mM Pb^2+^ solution.

The absorbance ratio with respect to time provides further insight into the capabilities of this detection system. Within 10 min, our system is able to detect the presence of Pb^2+^ by a quick and clear change in color as reflected in the spectra in Figure 3a,b. This provides the user with a rapid detection system using an extremely stable control as a foundation to compare the color change to. The absorbance values of GSH-GNPs upon the addition of 120–40 µL of 0.05 mM Pb^2+^ reached a maximum at 10 min, whereas the values of the other concentrations (30–10 µL) kept increasing. Upon the addition of 120 µL of 0.05 mM Pb^2+^, the system responded within seconds, indicating that the aggregation response of GSH-GNPs is highly dependent on the concentration of Pb^2+^. This result represents the fast performance of this probe for the detection of Pb^2+^. A visual time study to see how quickly we can detect Pb^2+^ at varying concentrations was conducted. In Figure 3c, we have shown the images depicting the progression of time and volume of 0.05 mM Pb^2+^ from 0 to 10 min. Within 10 min, this system provides visual proof of its ability to detect 25 µM Pb^2+^. Absorbance data provide further insight into the system’s capability to detect lower concentrations of Pb^2+^.

Determining the MDC of this system is dependent upon the linear regression of the relationship between the absorbance ratio and concentration. Such a relationship is used as a basis to quantify Pb^2+^ in aqueous solutions. The SPR peaks of the GSH-GNPs at 660 and 500 nm are related to the quantities of dispersed and aggregated GSH-GNPs, respectively. Therefore, we used the ratio of the values of absorbance at 660/500 (A_660/500_) to express the molar ratio of aggregated and dispersed GSH-GNPs. A linear calibration curve (R^2^ = 0.737) was observed from the linear regression of absorbance value versus concentration, Figure 3b. A system exhibiting an R^2^ value of 0.98 or higher would be ideal as this indicates its ability to quantitatively determine the Pb^2+^ concentration present in the system. From this, we suggest that this probe has the capability to be used for detecting Pb^2+^, but further optimization is needed in order to use this as a quantifying process.

**Figure 4:**
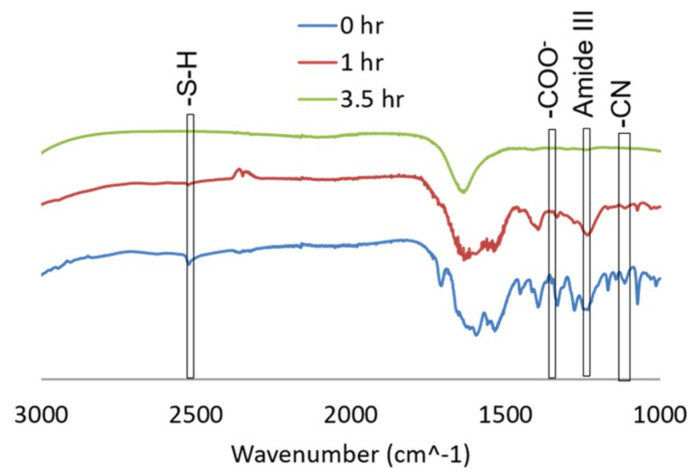
FTIR absorbance spectra of the glutathione peptide as a function of the accelerated heat stress exposure time. Reprinted from ref. [24].

The persistent attachment of glutathione was achieved through FTIR-ATR spectroscopy, as shown in Figure 4. The characteristic thiol functional group consisting of a hydrogenated sulfur is present in the GSH sample in its original form. A COO- absorbance peak was observed within the non-denatured GSH samples. As the GSH was heat stressed (shown in Figure 4), 10 µL aliquots were removed and the absorbance spectrum was monitored as a function of the exposure time. Figure 4 shows the decrease in the characteristic SH and COO- peaks as a function of the applied heat stress. After 1 h of degradation, the thiol and carboxylic acid groups decrease in intensity until the peaks are no longer distinguishable at 3.5 h of heat stress. This shows that the glutathione was attached to -COO^-^ binding sites.

**Figure 5:**
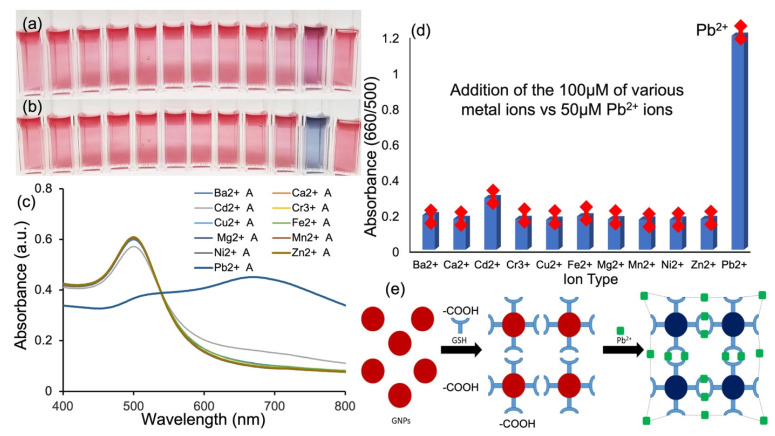
Detection of various ions at (**a**) 0 min and (**b**) 10 min. (**b**) From left to right: 100 µM Fe^2+^, Zn^2+^, Ba^2+^, Ni^2+^, Ca^2+^, Cr^3+^, Mg^2+^, Mn^2+^, Cu^2+^, Cd^2+^, 50 µM Pb^2+^, and the control. (**c**) Absorption spectra comparing control with 100 µL of various metal ions vs. 50 µM Pb^2+^ (all metals were incubated with the control for 10 min). (**d**) The absorbance values (A_660/500_) of the control upon addition of 100 µM of various metal ions vs. 50 µM Pb^2+^ ions and (**e**) schematic representation of the progression of GNPs after functionalization with GSH and the detection of Pb^2+^ through the formation of a coordination complex.

Several commonly existing metal ions were tested, including Fe^2+^, Zn^2+^, Ba^2+^, Ni^2+^, Ca^2+^, Cr^3+^, Mg^2+^, Mn^2+^, Cu^2+^, and Cd^2+^. The image below (Figure 5a,b) illustrates the GSH-GNPs in the presence of 100 µM of the various metal ions and 50 µM Pb^2+^. The results demonstrated that the other metal ions have no obvious effect on the system as compared to Pb^2+^. The Cd^2+^ ion, however, did show a mild effect on the control, as shown in Figure 5c–e, but nowhere near as much as Pb^2+^. However, this technique is also capable of detecting trace amounts of Cd^2+^ heavy metal, which is only 25% sensitive to Pb^2+^ ions due to poor binding of Cd^2+^ ions to -COO^-^ binding sites. Absorbance values (A_660/500_) of the control after the addition of the metal ions do not compare to those of the system with Pb^2+^ ions, as shown in Figure 5d, demonstrating the system’s high selectivity of Pb^2+^ over various common metals found in water, consistent with a recent report [25]. GSH has two free –COOH groups (shown as the ends of the Y-shape molecules in the schematics of Figure 4e) and a –NH^2^ group to provide a hydrophilic interface and a handle for further reactivity with heavy metal ions. The -NH^2^ group is protonated to -NH^3+^ and, as a result, -COO^-^ is the only binding site, which is known to bind strongly to Pb^2+^, as discussed earlier. Metal ions like Fe^2+^, Cd^2+^, and Zn^2+^ are known to bind to the amino group in GSH, but the amino group is made unavailable. The absorbance values at 660/500 (A_660/500_) express the molar ratio of aggregated and dispersed GSH-GNPs, and the linear calibration curve shows an R^2^ value of 0.98 or higher, indicating its ability, stability, and accuracy (with an error bar of less than 2%, Figure 5d) to quantitatively determine the Pb^2+^ concentration present in the system. The measurements were repeated at least three times and are highly reproduceable. Pb^2+^ ion detection in water remains a potentially important global topic [26], as several procedures have recently been reported [27,28].

## 4. Summary

We have produced a simple, cost-effective, quick, and portable detection method using a GSH-GNP-based colorimetric probe that allows rapid, real-time detection of Pb^2+^ in just 10 min. The experimental results show the strength of this system in terms of selectivity and the capability of improving the sensitivity to Pb^2+^ in aqueous solutions. Several commonly existing metal ions such as Fe^2+^, Zn^2+^, Ba^2+^, Ni^2+^, Ca^2+^, Cr^3+^, Mg^2+^, Mn^2+^, and Cu^2+^ have no obvious effect on the system compared to the Pb^2+^ ions as low as 50 µM. Cd^2+^ ions, however, did have a mild effect on the control, but nowhere near as much as Pb^2+^. However, this technique is also capable of detecting trace amounts of Cd^2+^ heavy metal, which is only 25% sensitive to Pb^2+^ ions due to the poor binding of Cd^2+^ ions to -COO^-^ binding sites. We believe this method may offer a faster approach for the detection of Pb^2+^ in aqueous biological and environmental samples for practical applications. Our method offers a solid proof-of-concept with proven experimental evidence for the rapid and cost-effective detection of Pb^2+^ toxic pollutants in water.
